# Level of modern health care seeking behaviors among mothers having under five children in Dangila town, north West Ethiopia, 2016: a cross sectional study

**DOI:** 10.1186/s13052-018-0503-z

**Published:** 2018-05-29

**Authors:** Amare Belachew Dagnew, Tilahun Tewabe, Rajalakshmi Murugan

**Affiliations:** 1 College of Medicine and Health science, Bahir Dar University, Bahir Dar, Ethiopia; 2 College of Health Sciences School of Allied Health Sciences, Addis Ababa University, Addis Ababa, Ethiopia

**Keywords:** Modern health care seeking behavior, Under five children, Dangila, North West Ethiopia

## Abstract

**Background:**

Health seeking behavior is an action taken by an individual who perceive to have a health problem. In most developing countries including Ethiopia the health of the children is strongly dependant on maternal health care behavior. Most childhood morbidities and mortalities are associated with low level of mothers health care seeking behavior. Therefore, the objective of this study was to assess level of modern health care seeking behavior among mothers having under five children in Dangila town, North West Ethiopia.

**Methods:**

Community based quantitative cross-sectional study was conducted from April 15 to May 15, 2016. Systematic random sampling technique was used to select study participants. A total of273 mothers with children less than five years were included in this study. The data was collected from all five Kebeles using interviewer administered questionnaire. Descriptive and inferential statistics were used to present the data. Both bivariate and multivariate logistic regression analyses were used to identify factors associated with level of modern health care seeking behavior.

**Results:**

Prevalence of modern health care seeking behavior was 82.1%. Age of mothers (AOR = 2.4(1.1, 5.3), age of the child (AOR = 6.7(2.8, 22.2), severity of illness (AOR = 5.2(1.2, 22.6) and family number (AOR = 6.4(2.1, 20.2) were predictors of modern health care seeking behavior among mothers.

**Conclusions:**

Majority of the mothers preferred to take their children to modern health care when they got illness. Age of children, age of mother, number of family and severity of illness were the determinant factors for modern health care seeking behavior. Therefore, health care services should be strengthened at community level through community integrated management of childhood illness, information, education communication / behavioral change communication strategies to improve mothers health care seeking behaviors.

## Background

Globally, 6.3 million children under the age of five years died each year. Sub-Saharan countries are among the regions where under five mortalities are highest [1].

The Ethiopian demographic health survey (EDHS 2011) showed under five mortality rates were higher among children from poor and non educated families. The risk of child death was higher in childrens who have uneducated mother [2].

Although modern health care interventions have the potential to substantially reduce childhood mortalities, a large number of children in developing countries died without ever reaching in health care facilities. Inability to recognize potentially life threatening conditions and pluralistic care seeking practices were the factors that make mothers to delay in seeking modern care timely. This delay affected child health significantly and leads to complications that make the medical care less effective [3].

Treatments of common childhood illness like diarrhea, malaria and pneumonia are usually very effective if the care is sought on time. However, to implement ongoing programs and to facilitate appropriate care remained as a challenge now a days [4].

Therefore, the morbidity and mortalities from these diseases can be reduced when care is sought early. The ability of caregivers to recognize and seek appropriate care of childhood illnesses is instrumental in reducing childhood deaths. Appropriate medical care seeking can prevent a significant number of childhood deaths and complications [5, 6].

Therefore, the purpose of this study was to assess mother’s modern health care seeking behavior for childhood illness in Dangila town, North West Ethiopia.

## Methods

Community based quantitative cross sectional study was conducted from April 15 to May 15, 2016. The study was conducted in Dangila town, Awi zone, Amhara Regional State, Ethiopia. It is located 485 km Northwest from Addis Ababa and 78 km from Bahir Dar, the capital city of Amhara region. The town has a total of five kebeles. It has a total of 5, 256 households and 30, 147 peoples and 1937 were under five children. There is a health center and two private clinics in the town.

The sample size was calculated using single population proportion formula by considering the following assumptions: prevalence (P) 73%, which is the prevalence of health seeking behavior in Bahir Dar town, North West Ethiopia [7], confidence level (CL) 95%, margin of error (d) 5% and after considering 10% non response rate the final sample size became 273. Using systematic random sampling technique, by selecting one from every seven households, a total of 273 households with under five children were selected. The first house hold were determined using lottery method which was three..

A structured interviewer administered questionnaire was used to collect data from mothers with under five children. It was constructed by adopting from previous research done on similar topics and modified accordingly. Four diploma nurses were recruited as data collectors and two Bachelor of Science nurses were recruited as supervisors. All data collectors and supervisors were oriented and trained on how to interview and record the data one day before the survey.

The collected data was checked manually for completeness and consistencies. Then it was coded, entered in EPI Info version 3.5.3 and exported to SPSS version 20 for analysis. Descriptive statistics was used to summarize the sociodemographic characteristics’ of the study participants and level of modern health care seeking behavior. To identify factors associated with mothers modern health care seeking behavior binary logistic regression analysis was carried out. Strength of association was measured using odds ratio and 95% confidence interval. Statistical significance was declared at *P* value < 0.05.

### Ethics approval and consent to participate

Ethical clearance was obtained from AAU, department of nursing and midwifery research committee. Each study participant was adequately informed about the objective of the study, anticipated benefits and risk of the study by their respective data collector. Verbal consent was obtained from study participants for protecting autonomy and ensuring confidentiality .

## Results

### Socio demographic characteristics

In this study, 273 mothers were participated. Above one third 102 (37.4%) of mothers were within the age group of 25 to 29 years with mean age of 28 years. About 66 (24.2%) mothers were unable to read and write, 220 (80.6%) were married and 134 (49.1%) were house wife’s. Regarding the average household income, about99 (36.3%) earn greater than 2000 Ethiopian birr per month. Most249 (91.2%) mothers had less than or equal to five family members. About 229 (83.9%) mothers had one under five children in the house. With regard to youngest child, 146 (53.6) were females and 190 (69.6%) were above one year (Table 1)**.**

**Table 1:** Socio demographic characteristics of mothers/caregivers and under five children are in Dangila town, Northwest Ethiopia, 2015

Variable	Response	Frequency (*N* = 273)	Percent (%)
Age of mothers/caregivers	< 25 years	55	20.1
	25–29 years	102	37.4
	30–34 years	65	23.8
	≥35 years	51	18.7
Age of child	≤1 years	83	30.4
	> 1–5 years	190	69.6
Sex of children	Male	127	46.5
	Female	146	53.5
Marital status of mothers.	Married	220	80.6
	Divorced	43	15.8
	Widowed	10	3.7
Religion of Mothers/care givers.	Orthodox	220	80.6
	Muslim	53	19.4
Ethnicity of mothers/ caregivers	Amhara	173	63.4
	Others^a^	100	36.6
Educational status	Unable to Write and read.	66	24.2
	Read and write	37	13.6
	Primary school	40	14.7
	Secondary school	58	21.2
	College and above	72	26.4
Occupation of mothers/ care givers.	House wife	134	49.1
	Government work	48	17.6
	Merchants	62	22.7
	Labor worker	29	10.6
Monthly income.	≤500	3	1.1
	501–1000	54	19.8
	1001–1500	56	20.5
	1501–2000	61	22.3
	> 2000	99	36.3
Family members	≤5	249	91.2
	> 5	24	8.8
Number of under five children	1	229	83.9
	> 2	44	16.1

^a^Tigray, Awe, Oromo

### Health seeking behavior

The overall four weeks prevalence of common childhood illness complained by mothers were: cough 70(26%), fever 65(24%) and diarrhea 55 (20%). Among 224 mothers, 177 (90%) of mothers made decision for seeking medical care by their own when their child got illness. While for 17(7.6%) cases the decision was made by their husband. The overall prevalence of modern health care seeking behavior of mothers was 82.1%. The main reasons mentioned for not seeking modern health care were: perceiving as illness is mild (24.5%), thought as sickness is incurable (16.3%), shortage of money (16.3%) and long period of waiting for medical services (14.3%) (Table 2).

**Table 2:** Proportion of common childhood illness among mothers/caregivers with under five children’s in Dangila town, Northwest Ethiopia, 2015

Variable		Frequency (*N* = 273)	Percent (%)
Complains of mothers/care givers.	Cough	70	26
	Difficulty of breathing	31	11
	Fever	65	24
	Diarrhea	55	20
	Ear infection	8	3
	Two and above symptoms	44	16
Decision makers for medical treatment	Mothers	177	79.0
	Fathers	17	7.6
	Grand parents	5	2.2
	Both mothers and fathers.	25	11.2
Distribution of health seeking behavior among mothers/care givers	Treat with Hole water	3	1.1
	Self treatment at home	3	1.1
	Take to traditional treatment	8	2.9
	Did nothing	12	4.4
	Treat the child by buying from pharmacy or drug sellers	23	8.4
	Take to private health facility	45	16.5
	Take to Government facility	179	65.9
Perceived illness stayed for medical care among mothers/care takers	On the same day of child hood illness	62	22.8
	After one days of child hood illness	126	46.4
	After two days of child hood illness	48	17.4
	After three days of child hood illness	37	13.4
Main reasons of mothers/caregivers for not seeking medical care	No treatment for sickness	6	2
	Cost of medical care	11	4.1
	No trust on health providers competency	17	6.1
	Lack of time	17	6.1
	Thought it get better by it self	28	10.2
	Don’t get immediate care / wait for several times for service	39	14.3
	Shortage of money	44	16.3
	Thought sickness is incurable	44	16.3
	Illness was mild	67	24.5

Regarding to perception of illness, nearly half 129 (47.3%) of mothers perceived their child illness was moderate, and about 156 (57.1%) mothers identifies the severity of illness by viewing different symptoms. From all, only 8(2.9%) mothers took their child to traditional healers for treatment and three of them used traditional treatment since they believed that disease doesn’t cure by modern health care (Table 3).

**Table 3:** Perception of mothers/caregivers about severity of common childhood illness among mothers/caregivers having under five children’s in Dangila town, Northwest Ethiopia, 2015

variable	Response	Frequency (*N* = 273)	Percent (%)
Perception of mothers about severity of illness.	Severe	125	45.8
	Moderate	129	47.3
	Mild	19	7
Ways of identification for the severity of disease.	By combined symptoms of the disease.	156	57.1
	My child refused to eat	59	21.6
	The illness continue for long time	58	21.2
Mothers seeking to traditional healers	No	265	97.1
	Yes	8	2.9
Main reasons for selecting traditional healers	Don’t get cure from medical care	3	37.5
	Treatment is effective	3	37.5
	Because family recommend to it	2	25

### Factors affecting mothers modern health care seeking behavior

In the bivariate logistic regression analysis: age of mother, occupational status, marital status, age of child, number of family and disease characteristics were statistically associated with modern health care seeking behavior.

In final multivariate analysis: age of the mother, age of the child, number of family numbers and perceived severity of illness were significantly associated with mothers modern health care seeking behavior.

Maternal age greater than or equal to 28 years were almost 2.4 times more likely to seek modern health care than those mothers less than 28 years [AOR = 2.4(1.1, 5.4)]. Mothers who had children less than one years of age were 6.7 times higher to seek modern health care than a mothers who have a child greater than one year’s [AOR = 6.7(2.9, 22.2)] . Mothers who have less than or equal to five family members were almost 6.4 times more likely to seek modern health care than mothers who have greater than five family members [AOR = 6.4(2.1, 20.2)] and mothers who perceive as their child illness was severe were five times higher to seek modern health care than mothers who perceive the illness was mild [AOR = 5.2(1.2, 22.6)] (Table 4).

**Table 4:** Factors that affect Health seeking behavior of mothers/care givers among children’s having common childhood illness using bivariate and multivariate logistic regression analysis model, in Dangila town, Northwest Ethiopia, 2015

Variables	Response	Yes	No	COR (95% CI)	AOR (95% CI)
Mother’s age	< 28	94(34.4)	30(11)	1	
	≥28	130(47.6)	19(7)	2.2(1.2–4.1)	2.4(1.1, 5.4)*
Age of children’s	≤1 years	79 (28.9)	4(1.5)	6.1(2.1–17.7)	6.9(2.9, 22.2)*
	> 1–5 years	145(53.1)	45(16.9)	1	
Marital status of mothers	Married	189(69.2)	31(11.4)	4.1(1.1, 15.2)*	
	Divorced	29(110.6	14(5.1)	1.4(0.3, 5.7)	
	Widowed	4(1.5)	6(2.2)	1	
Number of family members	≤5	210(76.9)	39(14.3)	3.8(1.6, 9.3)	6.4(2.1, 20.2)*
	> 5	14(5.1)	10(3.7)	1	
Occupation of mothers/caregivers	House wife	110(40.3)	24(8.8)	2.1(0.8–5.1)	
	Government work	44(16.1)	4(1.5)	4.9(1.4–18.0)	
	Merchant	50(18.3)	12(4.4)	1.9(0.1–5.1)	
	Labor worker	20(7.3)	9(3.3)	1	
Severity of illness	Sever	118(43.2)	7(2.6)	6.0(1.7–21.5)	5.2(1.2, 22.6)*
	Moderate	92(33.7)	37(13.6)	0.888(0 .3–2.6)	
	Mild	14(5.1)	5(1.8)	1	

*Statistically significant (*p* ≤ 0.05) on multi-variant logistic regression analysis

## Discussion

In this study 82.1% mothers were seek modern health care when their child got illness. This finding is similar to a study done in North West Ethiopia 84.4% mothers were seek modern care [7], higher than a study done in Yemen 51.4% [3]. However it is lower with a study done in Oromiya region Ethiopia 87% [8] and urban slum 90% [9]. This difference may be due to differences in socio demographic characteristics like: educational status, culture, economical status.

The least frequent action in this study was self treatment at home and treatment with holy water which accounts 1.1%. This finding was similar to study carried out in North west Ethiopia only 0.4% of them treat children illness at home [7] and lower than a study done in Nigeria 34.3% of them were treated at home [10]. This difference may be due to the presence of health package policy in Ethiopia like: expanding health extension programs, coordinated maternal and child health service and health education packages have been given at large.

In this study 79% of mothers were decision makers for seeking medical care by their own and the mean length of stay for treatment was two days. This result is similar to study done in Oromiya region Ethiopia [8] mean length of stay for medical treatment is two days, slightly faster than study results in Yemen most of mothers seek medical care after three days of onset of illness [3]. This could be due to differences in sociodemographic characteristics and health care seeking behavior. This is also related to expansion of urban health extension programs that creates awareness on early health seeking behavior.

Age of mother is one predictor of modern health care seeking behavior. Studies done in Nigeria and Ethiopia [7, 11] had reported a positive relationship between maternal age and health care seeking behavior, which is in agreement with the present study. This may be due to as the age of mother increases they will get experiences that help them to easily identify illness and seek medical care.

Mothers who had infants were seven times higher to seek modern care than those mothers who have older children. This finding is comparable to study done in Kenya, health care seeking was highest in the youngest age group (62.9%) and slowly declined thereafter for older groups 42.5% [12] and in a study conducted in North West Ethiopia [7]. This may be due to young infants were highly vulnerable to common childhood illness than elder ones and makes mothers to seek modern care easily.

Those mothers who have less than or equal to five family members were around six times higher to seek medical care than to those who have greater than five family members. This result is in line with a study in India those residing in joint family structures are predicting health seeking behaviors [13]. This may be related to low economy of the family and shortage of money for medical treatments.

In the current study, mothers who perceived illness was severe were five times more likely to seek medical care than those who perceive the illness is mild. This finding is similar to a systemic review done in developing countries, study carried out in Yemen and Kenya [3, 12, 14]. This difference may be due to mothers belief of illness will improve by itself and wait until their child show other symptom.

In this study, the main reasons for not seeking modern care were: percived illness was mild 12 (24.5%), thought sickness is incurable by medicine 8(16.3%) and shortage of money (2%) and this is an agreement with studies in Yemen illness was mild (39%), illness do not need medical treatment (31%) but contrasts to studies conducted in Amhara region: the disease did not need treatment(31.9%), bought drugs from drug vendors (27.2%), perception of the disease resolves by itself (56.7%), lack of money 24% [7, 15].

## Conclusion

The overall health seeking behaviors of mothers who have under five children was 82.1%.Age of the mother, age of the child, family number and perceived severity of illness were the significant factors for health seeking behavior. Recommendation to improve health seeking behavior include: informing the district health office to encourage family planning, educate, support and counsel the community at large and particularly to the women regarding the importance of seeking medical care. Health education about childhood illness in religious places, in schools and in meetings to increase awareness of mothers modern health care seeking behavior.

### Limitations

A limitation of this study was that it assessed only the quantitative aspects of level of modern health care seeking behavior among mothers having under five children. Qualitative study was not incorporated.

## Abbreviations

AOR: Adjusted Odds Ratio
COR: Crude Odds Ratio
EDHS: Ethiopian Demographic Health Survey
FMOH: Federal Ministry of Health
HSB: Health Seeking Behavior
UMR: Under five Mortality Rate
UNICEF: United Nation International Children’s Emergency Fund
WHO: World Health Organization
